# A Survey on Wellness and Its Predictors Amongst Fiji High School Students

**DOI:** 10.3389/fpubh.2021.671197

**Published:** 2021-05-10

**Authors:** Latileta Odrovakavula, Masoud Mohammadnezhad, Sabiha Khan

**Affiliations:** School of Public Health and Primary Care, Fiji National University, Suva, Fiji

**Keywords:** wellness, high school students, perceptions, predictors, Fiji

## Abstract

**Background:** Adolescent population face a number of health concerns which calls for objective and comprehensive assessment of their wellness during their critical development phase. This study aimed to determine adolescent wellness and its predictors amongst adolescents in secondary schools in Fiji.

**Methods:** This quantitative cross sectional study was conducted in four purposively selected schools in Suva and the greater Suva area, Fiji, between August and September, 2019. Students of Fijian nationality, enrolled into years 11-13 in the selected schools were purposively selected. A structured self-administered questionnaire was used to collect data on four dimensions of wellness including physical, emotional, social, and spiritual. Spearman's Rho correlation was conducted to test for associations. Descriptive and inferential statistical tests were applied to analyze the data by the SPSS software version 25. A *p*-value < 0.05 was considered significant.

**Results:** A total of 350 students participated in the study. Mean raw scores for wellness dimensions were as follows: physical = 51 (out of 60), psychological = 63 (out of 80), social = 42 (out of 50), and spiritual = 34 (out of 40). For overall wellness, two significant differences were observed: students of Fijian Itaukei descent (193.68 ± 14.2) and participants with a family income of $40,000-50,000 (199.08 ± 12.60) (*p* = 0.04) had a higher overall wellness score. There were three significant differences observed for psychological wellness dimension; Fijians of Indian descent (64.68 ± 9.30), participants enrolled into year 13 (64.68 ± 9.30) and those with a family income of $40,000-50,000 had higher psychological score. For social wellness, a significant difference was observed: Itaukei participants had higher scores (43.34 ± 4.42) when compared to other ethnic groups (*p* < 0.05). In terms of spiritual wellness, a significant difference was observed for ethnicity: Itaukei participants had a higher mean score (35.59 ± 4.26) when compared to other ethnic groups (*p* < 0.05). Strong correlations were observed for all dimensions of wellness.

**Conclusions:** Findings of this study highlighted different factors affecting adolescents' wellness in Fiji. It is recommended that health education and awareness program be carried out for developing adolescent wellness by considering these factors. It is also recommended that parental and family support are provided to adolescents.

## Background

According to World Health Organization (WHO) (2018), adolescents make up around 1.2 billion of the world's population (1). The adolescent years' poses as a critical phase of an individual's life and this is attributed to several factors. These include, the occurrence of rapid development and the need to apply decision making which influences habits that potentially influence adult onset of diseases and health issues (2). A range of health concerns are highlighted for adolescents. These range from premature death, illness, and injuries. In 2016 alone, more than 1.1 million adolescents died from preventable diseases or causes. Such statistics highlights the need to focus on of risky behaviors such as consumption of alcohol, tobacco use, physical inactivity, and unsafe sexual practices. These risky behaviors hinder adolescent growth and development and prevents them from reaching their full potential and optimal health (1). Health experts agree to the need of objectively and comprehensively assessing adolescent wellbeing at their critical development phase with the aim of progress toward healthy habits, influencing and supporting wellness (3).

Wellness is defined as, viewing individuals and their health from a holistic perspective that includes several dimensions involving the mind, body, spirit, and community interactions or social bonds (4). Previous literature supports wellness as a multi-dimensional concept consisting of interrelated components (5). For Fiji, the Wellness Fiji policy, considers seven dimensions, which includes social, spiritual, environmental, occupational, psychological, physical, and financial wellness (6). Literature also supports that for wellness to occur, there needs to be a balance between these dimensions (7).

Predictors and influences of adolescent wellness have been globally established. Review of literature indicates that for physical wellness, the predictors include physical activity, nutrition, and substance use (5, 8–12). Predictors for psychological wellness include self-concept and self-esteem (13–16). Family peers and school support have been observed to influence adolescent social wellness (13, 17–19). In terms of spiritual wellness, events such as experience of new social pressures, physical health/biological changes are observed to influence spiritual wellness (20).

The Fiji Ministry of Health and Medical Services (MoHMS) have seen the strengthening of the child health services and programs in the last 10 years with little investments in the health and well-being of adolescents (21). The adolescent and youth population in Fiji (10-24-Year-olds) comprises more than 25% of the total population (22). This is the population that will become adult citizens of the country and investing in them now, assures this group of young people have a chance to live to their fullest potential and obtain optimal health (6).

There is currently no data or research which specifically focuses on adolescent wellness in Fiji. To effectively implement interventions and action plans, data on adolescent wellness needs to be established. Therefore, the purpose of this paper is to determine adolescent wellness and its predictors amongst adolescents in secondary schools in Fiji.

## Materials and Methods

### Study Design and Setting

A cross-sectional study using a two stage cluster sampling was performed between August and September, 2019 in Suva, Fiji.

The first cluster considered the geographical location of the schools and these were divided to the four areas of Suva, Lami, Nasinu, and Nausori. The second cluster, considered the urban or peri-urban locations of the schools. A list from the Ministry of Education, Heritage and Arts (MoEHA) detailed the locations of the schools. From this school list, study sites were purposively selected to ensure there were representatives of both peri-urban and urban schools. It was also ensured that there was representation of ethnic, religious, and gender groups.

The study included 350 secondary school students enrolled in four purposively selected high schools in Suva and the greater Suva area, Fiji.

The following inclusion criteria were used; students of Fijian nationality enrolled into school grade or years 11-13 in the selected schools. An information sheet was provided to the participants, this briefly explained the study purpose. These consent forms were printed in three languages; English, Itaukei, and Hindi. Those with parental consent were allowed to participate in the study. These students were also provided assent forms to indicate their willingness to participate in the study. Students above the age of 18 were only provided assent forms.

### Data Collection Tool

A structured self-administered questionnaire was used to explore the perceptions of students toward adolescent wellness. Based on the four dimensions of physical wellness, psychological wellness, social, and spiritual wellness, questions were sourced from similar studies conducted on adolescent wellness (23, 24). Two questionnaires used by Spurr (2009) and Goodwin (2009) highlighted questions that were aligned to the objectives of this study and these questions were modified to the context of this study's setting (23, 24). Questionnaire by Spurr had 5 sections and 64 items whilst questionnaire by Goodwin (2009) had one overall section and 36 questions. Spurr (2009) had used the validated Adolescent Wellness Survey (AWS) created by Sharkey (1999) who assessed adolescent wellness amongst high school students in the United States. Goodwin (2009) had used the validated Perceived Wellness Survey (PWS). The original PWS had a total of 69-item questionnaire with six scales (25). Wellness related perceptions statements from these questionnaires were selected to develop the tool for this research quantitative tool. These questions were based on physical wellness, psychological wellness, social wellness, and spiritual wellness.

For this study, the questionnaire had six sections; section 1: demographic information (5 questions), section 2: perception of general wellness (5 questions), section 3: adolescent physical wellness (12 questions), section 4: adolescent spiritual wellness (8 questions), section 5: adolescent psychological wellness (16 questions), and section 6: adolescent social wellness (10 questions). A five-point Likert scale was used to measure adolescents' level of wellness. For the Likert scoring, strongly agree was given five points, agree had a score of 4, neutral scored at 3, disagree scored 2, and strongly disagree scored 1. For negatively worded statements, the codes were reversed with strongly disagree scoring the highest at 5 scores, to strongly agree scoring, 1. Each dimension of wellness had a minimum and maximum score. Physical wellness dimension had a maximum score of 60 and a minimum score of 12. Spiritual wellness had a maximum score of 40 and a minimum score of 8. Psychological wellness had a maximum of 80 and a minimum score of 16 and social wellness had a maximum score of 50 and a minimum score of 10. All questionnaires were printed in the English language as study participants were students of high schools where English is commonly used, developing the questionnaire in other languages was not required.

To ensure the study used good measurement tools, the validity and reliability of the data collection tool were checked. Validity considers the ability of tool to measure what it is supposed to measure and does so properly without including other factors (26). To test content validity, advice and reviews from academic staff that are considered experts in the field of wellness and health promotion was obtained. These academic reviews ensured questionnaire content was relevant and in alignment with the study's aim and objectives. The questionnaire was modified based on comments received.

Reliability test was also conducted for the survey questionnaire Likert scale items. Reliability is the degree in which a measurement tool or procedure is able to produce consistent results (26). Reliability of the item scale was calculated *via* Cronbach's alpha, indicating an acceptable internal consistency (α = 0.92).

### Study Procedure

Upon ethical approval, endorsement was sorted from MoEHA Research and Development section. A list of secondary school located in Suva was obtained from MoEHA. This enabled the selection of the schools to participate in the study.

Once schools were selected, arrangements were made for school visits; this allowed the researcher to conduct a courtesy visit to school management, seeking student and teachers' participation. Upon the receipt of approval, a focal person was identified for communication purposes. A meeting with the school's appointed focal staff was then conducted for further arrangements and briefing about the study importance, its aims and objectives. Through the focal staff, the rest of the academic staff were briefed about the study details and their role in the study participants' recruitment. The school focal person was requested to provide data collection dates for the school.

Students who met the inclusion criteria were invited to fill in the self-administered structured questionnaire. Time utilized to fill questionnaires was ~20-30 min. Clarifications on the questionnaire was provided when needed. Students were advised to submit their filled questionnaires after completion.

### Statistical Analysis

Data was exported and used in Microsoft Excel for data cleaning; cleaned data was imported back into Statistical Package for the Social Science (SPSS), version 25 for analysis. Due to the non-parametric nature of the data, Spearman's Rho correlation was conducted to test for associations. To examine the differences of means amongst the independent variables, Anova and Kruskal-Wallis *H*-test were used. Analysis of Variance (ANOVA) was conducted for parametric data and Kruskal-Wallis *H*-test was conducted for non-parametric data. A *p*-value < 0.05 was considered significant.

### Ethical Considerations

The study was approved by College of Medicine, Nursing and Health Sciences' (CMNHS) Health Research Ethics Committee (CHREC) at Fiji national University (FNU). Facility approval was endorsed by the Research and Development section of the Ministry of Education, Heritage and Arts (MoEHA). Consent forms to obtain a parent or guardian approval were provided to students, below the age 18 years.

## Results

### Demographic Characteristics of Participants

A total of 400 questionnaires were distributed, the study had a response rate of 87.5% with 350 questionnaire completed. Table 1 below, summarizes the socio demographic characteristics of participants. Participants' age, ranged from 16 to 20 years with the mean age of 17.5 (SD = +0.9). For this study's participants, more female students (60.9%) were part of the survey compared to male students (39.1%). In terms of ethnicity, there were more Fijians of Itaukei descent (58.6%) compared to other races. Most of the participants were enrolled into the Year 12 level (46%). Income levels provided by the students indicated that most of the students' family earned below $5,000 (23.4%) and between $10,000-20,000 (22%) annually.

**Table 1:** Socio-demographic characteristics of participants.

**Characteristics**	**n (%)**
Age (Mean ± SD)	17.5 ± 0.9
**Gender**	
Male	137 (39.1)
Female	213 (60.9)
**Age**	
16 years old	87 (24.9)
17–18 years old	239 (68.3)
19–20 years old	24 (6.9)
**Ethnicity**	
Itaukei	205 (58.6)
Indo-Fijian	118 (33.7)
Others	27 (7.7)
**Education Level**	
Years 11	111 (31.7)
Years 12	161 (46.0)
Years 13	78 (22.3)
**Annual Family Income (FJD)**	
Below $5,000	82 (23.4)
$5,000–10,000	57 (16.3)
$10,000–20,000	77 (22.0)
$30,000–40,000	54 (15.4)
$40,000–50,000	25 (7.1)
Above $50,000	24 (6.9)

### Level of Wellness

The mean adjusted overall wellness score was 188 with the range of 56-219. Mean raw scores for wellness dimensions were as follows: physical = 51 (out of 60), psychological = 63 (out of 80), social = 42 (out of 50), and spiritual = 34 (out of 40). In terms of overall wellness, nearly half of the participants (48.3%) had very high wellness scores. About 26% of participants scored high-level scores while another 25.7% had low scores. In terms of physical wellness, the majority of the participants scored very high scores 48.3% while 23.7% scored high scores and around 28.0% had low scores.

Similar results were found for psychological wellness; nearly half of the participants (49.7%) had very high wellness scores. About 26% of participants scored high level of scores while another 23.4% had low scores. For social wellness, 40.3% scored very high scores, with 30.9% scoring high scores and 28.9% scored low scores. In terms of spiritual wellness, 48.6% scored very high scores, 22.6% scored high scores, and 28.9% scored low scores. Table 2 presents participants level of wellness.

**Table 2:** Level of wellness.

	**Points scale**	**N (%)**	**Mean (±SD)**
**Overall Wellness**			188 ± 18
Low	56-179	90 (25.7)	
High	180-190	91 (26.0)	
Very High	191-219	169(48.3)	
**Physical wellness**			51 ± 5
Low	14-49	98 (28.0%)	
High	50-52	83 (23.7%)	
Very High	53-60	169 (48.3%)	
**Psychological wellness**			63 ± 7
Low	21-59	91 (26.0)	
High	60-63	85 (24.3)	
Very High	64-78	174 (49.7)	
**Social wellness**			42 ± 5
Low	10-40	101 (28.9)	
High	41-44	108(30.9)	
Very High	45-50	141 (40.3)	
**Spiritual wellness**			34 ± 4
Low	8-32	101 (28.9)	
High	33-35	79 (22.6)	
Very High	36-40	170 (48.6)	

### Predictors of Wellness

ANOVA analysis of overall wellness and each dimension of wellness scores, based on the study's independent variables are presented in Table 3.

**Table 3:** ANOVA of demographic variables, wellness composite, and wellness dimensions.

	**Overall wellness**		**Physical wellness**		**Psychological wellness**		**Social wellness**		**Spiritual wellness**	
	**mean ± Sd**	***p*-value**	**mean ± Sd**	***p*-value**	**mean ± Sd**	***p*-value**	**mean ± Sd**	***p*-value**	**mean ± Sd**	***p*-value**
**Gender**		0.952		0.374		0.068		0.775		0.081
Male	192.28 ± 17.89		50.94 ± 6.14		64.16 ± 6.98		42.76 ± 5.07		34.40 ± 4.20	
Female	192.15 ± 19.05		51.69 ± 5.29		62.17 ± 7.29		42.93 ± 5.52		34.79 ± 5.19	
**Age**		0.565		0.289		0.606		0.525		0.739
16 years old	192.45 ± 18.91		51.28 ± 6.08		63.42 ± 8.26		43.22 ± 5.08		34.51 ± 4.78	
17–18 years old	191.74 ± 18.73		51.29 ± 5.51		63.10 ± 6.91		42.70 ± 5.40		34.63 ± 4.88	
19–20 years old	195.96 ± 15.91		52.96 ± 5.17		64.62 ± 5.85		43.16 ± 5.80		35.20 ± 4.58	
**Ethnicity**		0.025^*^		0.652		<0.0001^*^		0.004^*^		0.0001^*^
Itaukei	193.68 ± 14.20		51.75 ± 4.48		63.00 ± 5.53		43.34 ± 4.42		35.59 ± 4.26	
Fijians of Indian Descent	191.63 ± 23.86		51.02 ± 7.13		64.68 ± 9.30		42.75 ± 6.19		33.16 ± 5.34	
Others	183.48 ± 19.77		50.44 ± 6.21		59.40 ± 6.38		39.74 ± 6.67		33.88 ± 4.91	
**Education Level**		0.340		0.433		0.042^*^		0.509		0.165
Years 11	190.95 ± 17.51		50.91 ± 5.53		62.97 ± 7.40		42.50 ± 5.01		34.56 ± 4.33	
Years 12	191.78 ± 21.21		51.48 ± 6.07		62.64 ± 7.80		42.85 ± 5.74		34.80 ± 5.49	
Years 13	194.85 ± 13.47		51.94 ± 4.81		65.07 ± 5.06		43.42 ± 4.93		34.41 ± 3.99	
**Family Income**		0.049^*^		0.268		0.012^*^		0.158		0.442
Below $5,000	187.90 ± 25.14		50.16 ± 7.55		61.68 ± 8.18		41.85 ± 6.55		34.20 ± 5.73	
$5,000–10,000	189.75 ± 15.08		52.16 ± 3.57		61.33 ± 6.81		42.15 ± 4.60		34.10 ± 4.64	
$10,000–20,000	194.58 ± 14.60		51.38 ± 5.51		64.75 ± 6.09		43.62 ± 4.89		34.83 ± 4.06	
$30,000–40,000	194.91 ± 15.75		52.19 ± 4.78		64.46 ± 6.51		43.38 ± 5.07		34.87 ± 3.88	
$40,000–50,000	199.08 ± 12.60		53.20 ± 3.54		65.08 ± 6.04		44.40 ± 4.50		36.40 ± 2.39	
Above $50,000	192.13 ± 18.70		50.63 ± 5.22		64.08 ± 9.31		43.04 ± 5.32		34.37 ± 7.23	

* *Non parametric tests were conducted*.

### Overall Wellness

Significant differences were observed for ethnicity (*p* = 0.02) where Fijians of Itaukei descent students had higher overall wellness scores (193.68 ± 14.2) as compared to those from other ethnic backgrounds. Participants with a family income of $40,000-50,000 had a higher overall wellness score (199.08 ± 12.60) when compared with those in other income categories and this was observed to be statistically significant (*p* = 0.04).

### Physical Wellness

There were no significant differences observed between the independent variables and physical wellness.

### Psychological Wellness

There were three significant differences observed for the psychological wellness dimension. This was observed for ethnicity, where Fijians of Indian descent had higher scores (64.68 ± 9.30) when compared to other ethnic groups; Itaukei (63.00 ± 5.53), and others (59.40 ± 6.38), (*p* < 0.01). Significant difference (*p* = 0.04) was observed for education level, participants enrolled in Year 13 had higher scores (65.07 ± 5.06) when compared with those enrolled in Year 12 (62.64 ± 7.80) and Year 11 (62.97 ± 7.40). In terms of family income, participants with a family income of $40,000-50,000 had a higher score (65.08 ± 6.04) when compared with those in other income categories (*p* = 0.01).

### Social Wellness

For social wellness, a significant difference was observed in terms of ethnicity, as Fijians of Itaukei descent participants, had higher scores (43.34 ± 4.42) when compared to other ethnic groups (*p* < 0.05).

### Spiritual Wellness

In terms of spiritual wellness, a significant difference was observed for ethnicity, as Fijians of Itaukei descent students had a higher mean score (35.59 ± 4.26) when compared to other ethnic groups (*p* < 0.05).

### Correlations Between Wellness Dimension Scores and Overall Wellness

Correlations between wellness dimensions and the overall wellness composite score are presented in Table 4. Strong or very strong correlations were observed for all dimensions: physical wellness score (*r* = 0.66, *p* < 0.01), psychological wellness score (*r* = 0.80, *p* < 0.01), social wellness score (*r* = 0.71, *p* < 0.01), and spiritual wellness score (*r* = 0.80, *p* < 0.01). These results show that there are strong positive relationships between the perceptions of each wellness dimensions and overall wellness.

**Table 4:** Correlations between wellness dimensions, wellness items, and overall wellness.

	**Overall wellness**	
	***r*-value**	***p*-value**
**Wellness dimensions**		
Physical wellness score	0.66	<0.01
Psychological wellness score	0.80	<0.01
Social wellness score	0.71	<0.01
Spiritual wellness score	0.80	<0.01
**Physical wellness statements**		
My weight affects my physical development.	0.19	<0.01
I eat a balanced diet.	0.29	<0.01
Physical activity is important.	0.29	<0.01
Drinking Kava affects my health.	0.37	<0.01
Marijuana affects my health.	0.40	<0.01
Sniffing glue or Solvents affects my health.	0.39	<0.01
Using drugs such as Cocaine, Heroin … affects my health.	0.40	<0.01
Drinking anything with alcohol affects my health.	0.41	<0.01
**Psychological wellness statements**		
I can answer the question “Who am I”.	0.54	<0.01
I always look on the bright side of things.	0.47	<0.01
I am able to learn from my challenges	0.47	<0.01
I feel competent in dealing with life challenges.	0.47	<0.01
I sometimes think I am a worthless individual.	0.10	<0.01
I don't understand what life is all about.	0.18	<0.01
I am uncertain about my ability to do things well in the future.	0.02	<0.01
I have grown up in an affectionate, accepting and loving family	0.52	<0.01
I believe my life has purpose right now	0.53	<0.01
My Family Cares for me	0.52	<0.01
My friends care for me	0.48	<0.01
I feel like I can make good decisions	0.53	<0.01
I like myself just the way I am	0.47	<0.01
**Spiritual wellness statements**		
Spirituality brings me a sense of hope meaning and purpose in life.	0.53	<0.01
Spirituality means having a sense of connectedness to a higher power.	0.51	<0.01
Spirituality teaches you what is right and what is wrong	0.58	<0.01
Spirituality enriches a person's quality of life.	0.55	<0.01
Spirituality is important	0.52	<0.01
**Social wellness statements**		
My family at home supports me.	0.53	<0.01
My parent/guardian is nurturing, warm and accepting.	0.57	<0.01
My parent/guardian takes interest in where I am, whom I am with, and what I am doing.	0.44	<0.01
I feel connected to my friends.	0.40	<0.01
I receive support from my teachers and school counselors.	0.49	<0.01
I feel that the people in my school care about me.	0.52	<0.01
I receive support from my youth group.	0.47	<0.01

Correlation analysis between physical wellness items and overall wellness produced positive, low, weak, and moderate levels of associations. These included perceptions related to weight, diet, and physical activity. A positive moderate correlation was observed for the perceptions related to smoking marijuana (*r* = 0.40, *p* < 0.01), using drugs (*r* = 0.40, *p* > 0.01), and drinking alcohol (*r* = 0.41, *p* < 0.01).

Correlations between all high self-concept variables were moderately associated with overall wellness. These included, the ability to answer the question of “who am I” and the perception of “I always look on the bright side of things” with both scoring (*r* = 0.54 and *r* = 0.47, *p* < 0.01), respectively. The belief that there is purpose to life had a moderate relationship with overall wellness (*r* = 0.53, *p* < 0.01).

For the statements related to psychological support from family and friends, moderate relationships were also observed. Growing up in an affectionate and loving family (*r* = 0.52, *p* < 0.01) and being cared for by friends (*r* = 0.48, *p* < 0.01), both had positive moderate associations with overall wellness.

In terms of spiritual wellness related statements, spirituality bringing a sense of hope [(*r* = 0.53, *p* < 0.01) and being is connected to a higher power (*r* = 0.51, *p* < 0.01)] are positively correlated with overall wellness. Moderate correlations were observed for both “spirituality teaches you what is right and what is wrong” (*r* = 0.58, *p* < 0.01) “spirituality enriches a person's quality of life” (*r* = 0.55, *p* < 0.01) and the belief that “spirituality is important” (*r* = 0.52, *p* < 0.01).

All of the statements pertaining to social wellness have significant moderate correlations with overall wellness. Students belief in, being supported from home, had a correlation coefficient of *r* = 0.53 (*p* < 0.01). Similar results were found for the item “My parent/guardian is nurturing, warm and accepting” (*r* = 0.57, *p* < 0.01). For items related to support from peers and school, all had moderate correlations.

## Discussion

The study findings indicated that the majority of the adolescents scored on the high or very high end of the wellness score scale. This was similar to a study done by Preskitt which looked at adolescent wellness amongst those aged 12–17 years. Scores computed from the survey observed that nearly 30% of students scored low levels of wellness and another, 26% scored high levels of wellness score, the majority, 48.3% scored very high scores (3).

Different levels of wellness exist may be attributed to various school programs such as physical education classes, extracurricular activities, schools visit by organizations' providing advocacy on health, support from family, from peers and teachers who take up the role of school counselors by default (27, 28). These activities boost adolescent wellness. A third of the participants had low scores. These could be attributed to the negative influence of factors such as lack of support, social and education expectations, and extensive use of social media. Similar results were found by Berk, the study attributed low level of wellness scores to social and academic expectations placed on adolescents (29).

The study found seven significant differences between wellness variables and the independent variables. Four significant differences were observed for ethnicity. Three of these findings saw Fijians of Itaukei decent students with higher scores when compared to those from other ethnic backgrounds for overall wellness, social and spiritual wellness. Petersen et al. in their study stated that the differences observed between ethnic groups could be potentially explained by socio cultural differences (30). This included Itaukei ethnic group having a collectivist ethos that is characterized by interdependence where communal needs take priority over individual needs. Such practice enhances social ties in the Itaukei communities creating a bond between relatives despite the distant relations; these are observed to be social networks of support that often extends a helping hand in hard times (31). In addition to social wellness, participants of Itaukei ethnic groups had higher scores for physical wellness and spiritual wellness as well. This was in contrast to the previously mentioned study conducted by Petersen et al., the study found students of Itaukei ethnicity had a low level of physical, emotional, social, and school functioning and wellbeing (30). The authors had attributed their findings to ethnic differences.

As results indicated, religion is considered sacred and important to Itaukei people. Literature also indicates this, stating that Christianity is considered core aspect of identity (32). This may potentially explain results of Itaukei participants having higher scores for spiritual wellness.

Another significant difference was observed for psychological wellness dimension and ethnicity where, Fijians of Indian descent had higher levels of psychological wellness when compared to other ethnic groups. Previous studies have found differences in ethnic background influenced psychological wellness (33, 34). These studies suggested that although it is essential to consider socio economic factors as confounders, ethnic identity has significant associations with mental health. Willard and McNamara had observed this significant association through their study and concluded that Fijians of Indian descent tend to focus more on internal mental states than Itaukei Fijians (32). This may potentially explain the significant difference of psychological wellness between the two ethnic groups.

The fifth significant difference was observed between psychological wellness and education level. Participants enrolled in the higher-class levels had higher scores when compared to those who were younger and enrolled into the lower classes. This was observed for all wellness dimensions and could be attributed to older adolescents being able to make sense of their development when compared to younger ones (35).

Two significant findings were associated with family income; overall wellness and psychological wellness. Participants with a family income of $40,000-50,000 had higher overall wellness and psychological scores when compared with those in other income categories. Literature indicated that low income is associated with lower wellbeing, lower life satisfaction and lower quality of life (36, 37). Income inequality still remains an issue for Fiji and is stated to be higher in the urban areas when compared to the rural areas (38).

Results of correlation analysis found strong or very strong correlations for all dimensions. These results show that there are strong positive relationships between the perceptions of each wellness dimensions and overall wellness. The results of the study suggested that an increased sense of wellness was strongly associated with higher dimensional scores.

For the physical dimension findings, results found that perceptions of weight, diet, and physical activity as important were significantly correlated with wellness. There were also significant moderate correlations between substance use and wellness; these suggested students may recognize the potential influence of weight, diet, and physical activity on health. This is essential to address the health issues associated with these risk factors amongst the adolescent population. Past studies done in Fiji show concerns of poor dietary patterns and the use of substances amongst adolescents (10, 39, 40).

Consistent with literature, significant correlations were also observed for psychological wellness dimensions. This study found significant relationships exist between positive self-concept, self-esteem related statements and wellness. Previous studies have shown that those with high self-esteem and self-concept tend to have better mental health, academic achievements and are less likely to do drugs (16, 41–43). There is evidence of the influence of self-concept and self-esteem and further tools are to be developed to evaluate the concepts further in Fiji.

This study found that receiving support from family and friends led to better psychological wellbeing. Other studies have found similar significant findings (13, 44, 45). These results suggest to stakeholders working with adolescent wellness, that family and friends are important influences of adolescent wellness and these relationships are to be considered. Significant findings were also observed for social wellness. The study found that family support, having a warm and nurturing parents or guardians are significantly correlated with higher sense of wellness. This is supported by other research (17).These findings suggest that adolescents view family and peers to influence their social wellness and adolescent programmes are to consider this.

Significant findings were also observed for spiritual wellness. The perception of spiritual wellness as important was moderately associated with spiritual wellness. This highlights a high sense of spiritual wellness as being important. Similar results were found for statements relating to the meaning of spiritual wellness. These findings may suggest what other studies have found. This include, having a higher sense of spiritual wellness leads to the practice of less risky behaviors such as consumption of drugs and alcohol (46, 47). This may be influenced by rules set as acceptable behavior in the spiritual or religious disciplines, which may strongly discourage use substance use, and deviant behavior (48). Other literature have also observed that higher sense of spiritual wellness was associated to coping mechanisms (46).

## Limitations

Findings of this research must be interpreted within the context of its limitation. Due to the setting and the nature of the study participants, timing of data collection was limited. Quantitative data tool used was a self-administered questionnaire that could possibly explain the high scores of wellness observed for the study. Due the study being cross sectional, the study is limited to high school students and findings may not be generalized to all adolescent population in Fiji. Study findings included only students and teachers, it would be ideal for future research to consider parents and guardians' perception as they are vital toward achieving adolescent wellness.

## Summary and Conclusion

The findings of this study are related to ethnicity, family income, physical activity, diet and body weight, support from peers, friends, and family, high self-concept and self–esteem, and spiritual wellness meanings. It is recommended that health education and awareness be carried out in terms of physical activity, diet, and body weight. It is also recommendedthat MoEHA research on best ways to increase parental engagement in schools. Apart from family, peer support can be a coping mechanism for adolescent and adolescent wellness programs are to consider peer support interventions. MoHMS and MoEHA have collaborated over the years to improve and encourage efforts in the school health programs. This collaboration is to continue, strengthened, and improved to allow MoHMS programs to be efficiently and effectively implemented in school settings. This collaboration is to ensure that wellness is reflected in the school health programs. Finally, there is more research effort needed to inform the wellness movement in Fiji. This study has added to the adolescent wellness pool of knowledge, however; research gaps remain. Further studies are needed to understand the broad concept of wellness and its several dimensions. The knowledge level, the perceptions, and practice of wellness amongst other population groups remain unanswered.

## Data Availability Statement

The raw data supporting the conclusions of this article will be made available by the authors, without undue reservation.

## Ethics Statement

The studies involving human participants were reviewed and approved by Fiji National University's (FNU) College Health Research Ethics Committee (CHREC). The patients/participants provided their written informed consent to participate in this study.

## Author Contributions

LO led the writing of the article and contributed to the data analysis and interpretation of the results. MM contributed to the technical aspects of the research and data analysis. SK led the data analysis process. All authors contributed to the article and approved the submitted version.

## Conflict of Interest

The authors declare that the research was conducted in the absence of any commercial or financial relationships that could be construed as a potential conflict of interest.
